# Mixed Metabolic and Respiratory Alkalosis: An Uncommon Presentation of Superior Mesenteric Artery Syndrome in an Adolescent Male Patient

**DOI:** 10.7759/cureus.103655

**Published:** 2026-02-15

**Authors:** Ahmad Mohammad, Mohamed K Said, Pavani Durga Kolluru, Asad Raza

**Affiliations:** 1 Internal Medicine, Hurley Medical Center, Flint, USA; 2 Cardiovascular Medicine, Mansoura University, Mansoura, EGY; 3 Internal Medicine, Shri B. M. Patil Medical College, Vijayapura, IND; 4 Internal Medicine, Lahore Medical and Dental College, Lahore, PAK

**Keywords:** adolescent, duodenal obstruction, gastrointestinal obstruction, metabolic alkalosis, pediatrics, respiratory alkalosis, strong’s procedure, superior mesenteric artery syndrome, wilkie’s syndrome

## Abstract

Superior mesenteric artery (SMA) syndrome is an uncommon cause of proximal intestinal obstruction resulting from compression of the third portion of the duodenum between the abdominal aorta and the SMA. Patients typically present with postprandial epigastric pain, bilious vomiting, and weight loss, and most exhibit metabolic alkalosis due to persistent loss of gastric acid. We report the case of a 16-year-old male child with progressive postprandial vomiting and significant nutritional decline who was diagnosed with SMA syndrome on the basis of reduced aortomesenteric angle and marked duodenal dilatation on imaging. Uniquely, the patient demonstrated a mixed acid-base disorder, combining metabolic alkalosis from chronic vomiting with respiratory alkalosis likely secondary to hypoxia-induced hyperventilation. The patient underwent Strong’s procedure with good postoperative recovery and resolution of symptoms. This case emphasizes the importance of recognizing atypical metabolic profiles in SMA syndrome, maintaining clinical suspicion in adolescents with chronic vomiting and weight loss, and intervening early to prevent complications.

## Introduction

Superior mesenteric artery (SMA) syndrome, also known as Wilkie’s syndrome, is an uncommon cause of proximal intestinal obstruction resulting from extrinsic compression of the third portion of the duodenum between the abdominal aorta and the overlying SMA [1]. This occurs when the normal aortomesenteric angle (typically 38-65°) and distance (10-28 mm) are significantly reduced, most often due to the loss of the retroperitoneal mesenteric fat pad. Patients typically present with postprandial epigastric pain, early satiety, nausea, bilious vomiting, and significant weight loss, creating a self-perpetuating cycle in which further malnutrition exacerbates the anatomical narrowing [2]. Although recognized for more than a century, SMA syndrome remains a diagnostic challenge due to its nonspecific symptoms and low prevalence, with a higher predilection for adolescent and young adult females. Delayed recognition risks complications such as gastric dilatation, electrolyte abnormalities, and, in severe cases, gastric perforation [3].

The objective of this case report is to highlight an uncommon clinical presentation of SMA syndrome characterized by a mixed acid-base disorder-specifically, the simultaneous occurrence of metabolic and respiratory alkalosis, a deviation from the typical isolated metabolic alkalosis associated with persistent vomiting. By documenting this case, we aim to enrich clinicians’ understanding of the diverse physiologic consequences of duodenal obstruction, underscore the importance of integrating imaging with biochemical assessment in establishing the diagnosis, and emphasize timely surgical intervention when conservative management fails. This case adds valuable insight into the atypical metabolic profiles that may arise in SMA syndrome and reinforces the need for heightened clinical suspicion in patients with chronic vomiting and unexplained weight loss.

## Case presentation

A 16-year-old male patient with no known comorbidities presented with a one-year history of intermittent postprandial vomiting, which had markedly worsened over the preceding two months. The vomiting occurred within 20-30 minutes after meals, was bilious, and contained partially digested food. Episodes were accompanied by epigastric pain, early satiety, and progressive reduction in oral intake. The patient reported significant unintentional weight loss over the past several months, though exact quantification was not available. There was no history of hematemesis, melena, dysphagia, chronic medication use, prior abdominal surgery, or known eating disorders. Family history was non-contributory.

On examination, the patient appeared thin, mildly dehydrated, and fatigued. His BMI, estimated from recorded height and weight, was below the fifth percentile for age, consistent with malnutrition. Vital signs revealed a pulse of 102 beats/minute, blood pressure of 108/70 mmHg, respiratory rate of 22 breaths/minute, and temperature of 36.8°C. Abdominal exam showed epigastric fullness, mild tenderness without guarding, and visible peristalsis. Bowel sounds were hyperactive. No organomegaly or lymphadenopathy was noted.

Initial laboratory investigations are summarized in Table 1. The patient demonstrated a microcytic, hypochromic anemia with low hemoglobin (12.9 g/dL), reduced mean corpuscular volume (MCV) and mean corpuscular hemoglobin (MCH), and elevated eosinophils and monocytes. Electrolyte values at admission were within acceptable ranges, though repeated episodes of vomiting placed him at risk for depletion. These hematological abnormalities were attributed to chronic nutritional compromise rather than a primary hematologic disorder.

**Table 1:** Summary of baseline complete blood count at the time of presentation, demonstrating microcytic indices and borderline anemia consistent with chronic nutritional compromise.

Parameter	Result	Units	Reference Range
Hemoglobin	12.9	g/dL	13–16
Red blood cell	4.85	×10⁶/µL	4–6.2
Hematocrit	36.4	%	42–51
Mean corpuscular volume	75.2	fL	80–100
Mean corpuscular hemoglobin	26.6	pg	27–32
Mean corpuscular hemoglobin concentration	35.4	g/dL	30–35
Total leukocyte count	4.7	×10³/µL	4–11
Platelets	241	×10³/µL	150–400
Monocytes	10.9	%	2–10
Eosinophils	4.5	%	0–4

**Figure 1:**
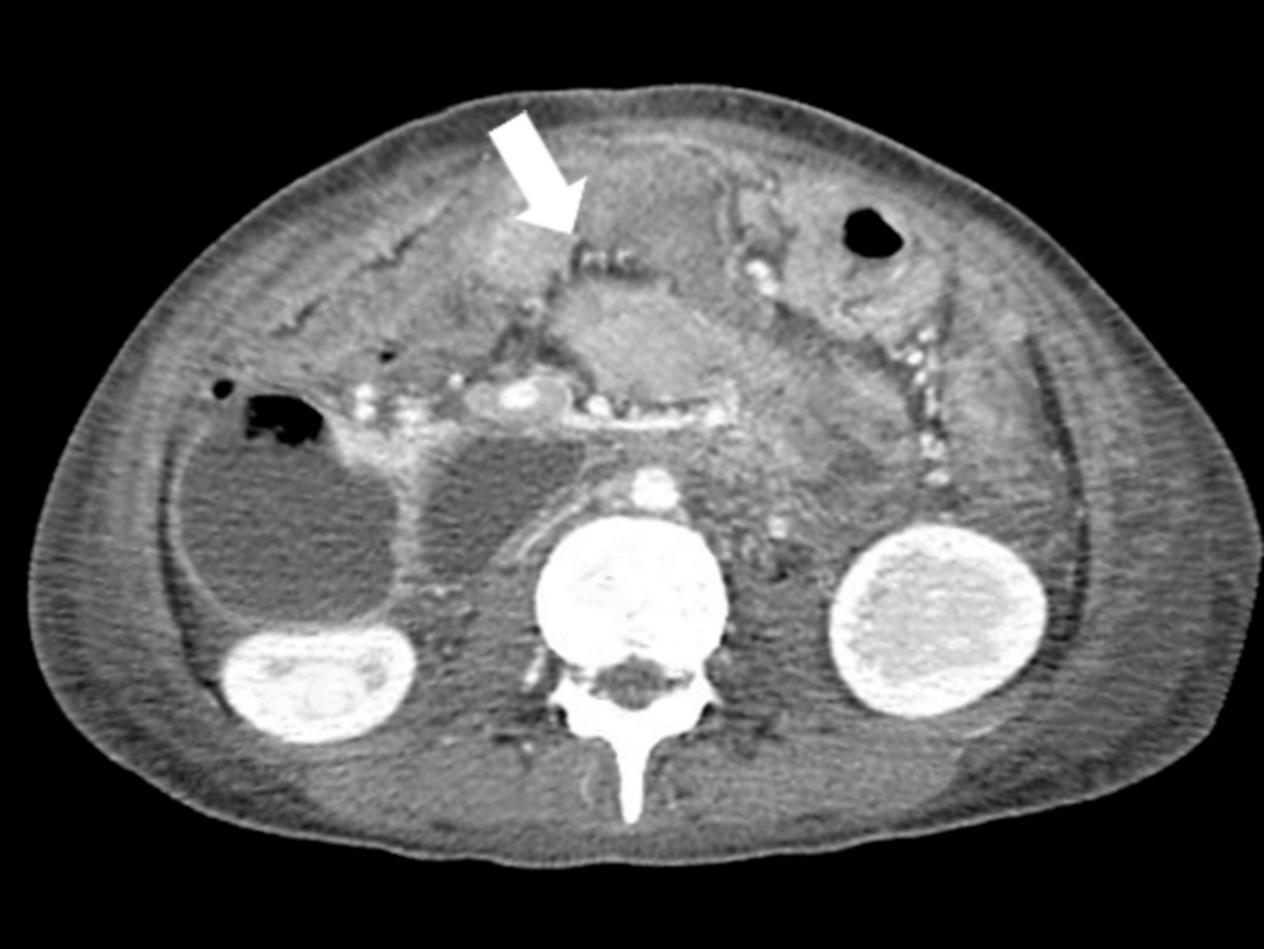
Contrast-enhanced CT scan demonstrating significant dilatation of the stomach and proximal duodenum due to extrinsic compression of the third portion of the duodenum. The arrow marks the site where the SMA compresses the duodenum against the abdominal aorta, consistent with SMA syndrome. SMA: superior mesenteric artery

An abdominal ultrasound revealed a markedly dilated stomach extending to the pylorus, with associated right-sided mild hydronephrosis. Due to concern for a proximal obstructive pathology, a contrast-enhanced computed tomography (CT) scan of the abdomen was performed. CT demonstrated a significantly reduced aortomesenteric angle measuring approximately 16°, with narrowing of the space between the SMA and abdominal aorta, resulting in compression of the third portion of the duodenum, as illustrated in Figure 1. This led to notable upstream dilation of the stomach and proximal duodenum. Other abdominal organs appeared unremarkable. These findings were strongly suggestive of SMA syndrome.

To further evaluate upper gastrointestinal anatomy, a barium swallow study was performed. Oral and pharyngeal phases of swallowing were normal, with no aspiration or reflux. Contrast passed through the esophagus and into the stomach without delay, but proximal duodenal distention was again noted, supporting a diagnosis of partial duodenal obstruction due to SMA compression.

Differential diagnoses considered included peptic ulcer disease with gastric outlet obstruction, congenital duodenal web, chronic pancreatitis, malrotation with intermittent volvulus, functional dyspepsia, and eating disorders. However, the distinctive radiologic picture of reduced aortomesenteric angle and duodenal compression strongly supported SMA syndrome as the primary etiology. The provisional diagnosis was superior mesenteric artery syndrome with progressive gastric and duodenal dilation.

Given the severity of symptoms, failure of nutritional optimization, and persistent obstructive physiology, the surgical team proceeded with Strong’s procedure. Through an upper midline incision, the duodenojejunal flexure was identified, and the ligament of Treitz was divided, allowing derotation and mobilization of the duodenum away from the narrowed aortomesenteric angle. This maneuver relieved the extrinsic compression without the need for bowel resection. An intraoperative nasogastric tube was placed for decompression.

Postoperatively, the patient was maintained *nil per os* (nothing by mouth) and provided intravenous fluids, electrolyte replacement, and analgesia. Nasogastric output gradually reduced, and bowel sounds returned by postoperative day 3, after which oral intake was slowly resumed. An arterial blood gas (ABG) was obtained immediately postoperatively, prior to full correction of the pre-existing metabolic derangements, and is summarized in Table 2. The ABG demonstrated an alkalemic pH with low partial pressure of carbon dioxide (pCO₂) and hypokalemia, consistent with a mixed metabolic and respiratory alkalosis that reflected the patient’s preoperative physiologic state, characterized by chronic vomiting-induced metabolic alkalosis and hypoxia-driven hyperventilation. These abnormalities improved during the subsequent postoperative recovery period with nutritional repletion and electrolyte correction.

**Table 2:** Postoperative arterial blood gas analysis pCO₂: partial pressure of carbon dioxide; pO₂: partial pressure of oxygen; HCO₃⁻: bicarbonate

Parameter	Result	Units	Reference Range
pH	7.416	—	7.35–7.45
pCO₂	31.4	mmHg	32–45
pO₂	62.4	mmHg	75–100
HCO₃⁻	28	mmol/L	22–26
Base Excess	+4	mmol/L	–2 to +2
Potassium	2.85	mmol/L	3.4–4.5
Sodium	134	mmol/L	135–145

The mixed alkalosis was attributed to chronic vomiting (causing metabolic alkalosis) and compensatory hyperventilation in response to hypoxemia (leading to respiratory alkalosis). Electrolyte abnormalities were corrected with intravenous supplementation. Over the following days, the patient’s vomiting subsided, abdominal discomfort improved, and oral intake increased. He was discharged in stable condition with instructions for nutritional rehabilitation and scheduled follow-up. At subsequent outpatient review, he reported progressive weight gain and complete resolution of postprandial symptoms.

## Discussion

SMA syndrome is an uncommon cause of proximal intestinal obstruction and remains challenging to diagnose due to its nonspecific symptoms. It results from a significant narrowing of the aortomesenteric angle, typically secondary to rapid or chronic weight loss that depletes the protective mesenteric fat pad. This anatomical change compresses the third portion of the duodenum and produces a characteristic constellation of postprandial epigastric pain, bilious vomiting, early satiety, and progressive nutritional decline [4]. In this case, the reduced aortomesenteric angle of 16° and marked proximal dilation on imaging confirmed the diagnosis.

Metabolic disturbances are common in SMA syndrome, largely driven by persistent vomiting. Loss of gastric hydrogen and chloride ions leads to a classic hypochloremic, hypokalemic metabolic alkalosis, further exacerbated by reduced intravascular volume and secondary hyperaldosteronism, which promotes renal potassium wasting [5]. This biochemical profile is frequently reported in SMA syndrome. Notably, however, our patient exhibited a mixed acid-base disorder, combining metabolic alkalosis with respiratory alkalosis. The respiratory component was likely due to tachypnea in response to mild hypoxemia, as reflected by the reduced arterial pO₂, although there was no radiographic or clinical evidence of aspiration pneumonia or intrinsic pulmonary pathology. This dual disturbance is rarely described and underscores the importance of evaluating the full acid-base spectrum in patients with chronic obstruction, particularly when symptoms or laboratory findings deviate from expected patterns [6]. Additionally, while the hematologic abnormalities were most consistent with nutritional compromise, the mild eosinophilia could also be seen in allergic or parasitic conditions, though no clinical evidence supported these etiologies in this case.

The hematologic findings in this case, including microcytosis, low hematocrit, and borderline anemia, are not intrinsic to SMA syndrome but likely reflect chronic malnutrition, iron deficiency, or micronutrient depletion resulting from prolonged vomiting. Mild monocytosis and eosinophilia may indicate nonspecific inflammation or early nutritional stress, although eosinophilia may also be seen in parasitic or allergic conditions; however, no clinical features suggested these alternative etiologies in this patient. These abnormalities underscore the systemic physiological burden imposed by untreated duodenal obstruction, especially in adolescents with increased metabolic demands [7].

Diagnosis of SMA syndrome requires high clinical suspicion, particularly in young patients with unexplained weight loss and recurrent postprandial symptoms. Contrast-enhanced CT remains the diagnostic modality of choice, enabling precise measurement of the aortomesenteric angle and visualization of duodenal compression [8]. Complementary studies, such as upper gastrointestinal contrast series, can demonstrate delayed transit and proximal dilation. Delay in diagnosis may predispose to serious complications, including gastric pneumatosis, portal venous gas, bezoar formation, or perforation.

Management initially focuses on nutritional rehabilitation to restore retroperitoneal fat and widen the aortomesenteric angle. When conservative therapy fails or obstruction is severe, surgical intervention becomes necessary. Strong’s procedure, as performed in this case, effectively relieves duodenal compression without anastomosis, whereas duodenojejunostomy remains the preferred definitive operation in many centers due to its high success rate. Both procedures aim to bypass or eliminate the point of duodenal compression and are selected based on the surgeon's expertise, anatomical considerations, and the patient's condition.

This case contributes to the existing literature by highlighting a rare mixed alkalosis pattern in SMA syndrome and underscores the need for comprehensive biochemical and radiologic assessment. Early recognition, appropriate imaging, and timely surgical intervention are essential to prevent complications and ensure full recovery. A limitation of this report is that only the CT scan image was available for documentation, although the imaging findings were sufficiently diagnostic to support the conclusions presented. Additionally, the timing of the postoperative ABG may introduce interpretive challenges, as the values likely reflected residual preoperative physiologic disturbances; this was clarified in the case presentation but remains an inherent limitation of retrospective case reporting.

## Conclusions

SMA syndrome is an uncommon but clinically significant cause of proximal intestinal obstruction, often presenting with vague gastrointestinal symptoms that can delay diagnosis and increase the risk of serious complications. This case underscores the importance of maintaining a high index of suspicion in adolescents with chronic postprandial vomiting, weight loss, and radiographic evidence of duodenal compression. The unusual finding of mixed metabolic and respiratory alkalosis highlights the need for comprehensive biochemical assessment, as deviations from expected metabolic patterns may signal additional physiological stress. Early recognition, appropriate imaging, nutritional optimization, and timely surgical intervention remain essential for preventing morbidity. Ultimately, clinicians should be aware that SMA syndrome can manifest with atypical metabolic disturbances, and prompt management can lead to complete symptom resolution and favorable long-term outcomes.

## References

[1] Abourak C, Oukassem S, Guennouni A, Bahha S, El Fenni J, Hassan EN (2025). A rare cause of high intestinal obstruction: Wilkie's syndrome associated with a suprarenal abdominal aortic aneurysm. Radiol Case Rep.

[2] Lurie A, Guntupalli L, Stoll V, Moon A (2023). Superior mesenteric artery syndrome masquerading as irritable bowel syndrome: a case report. Cureus.

[3] Hassan O, Sumbizi C, Kitua A, Gabone J, Fidaali Z, Ali A (2025). Superior mesenteric artery syndrome, an uncommon cause of gastric outlet obstruction: case report. Int J Surg Case Rep.

[4] Makary MS, Rajan A, Aquino AM, Chamarthi SK (2017). Clinical and radiologic considerations for idiopathic superior mesenteric artery syndrome. Cureus.

[5] Sur M, Hashmi MF (2024). Alkalosis. StatPearls [Internet].

[6] Park M, Sidebotham D (2023). Metabolic alkalosis and mixed acid-base disturbance in anaesthesia and critical care. BJA Educ.

[7] Chaudhry HS, Kasarla MR (2023). Microcytic hypochromic anemia. StatPearls [Internet].

[8] Maghraby GG, Elgendy H, Marzouk A, Awad E (2025). Vomiting as the storyteller in superior mesenteric artery syndrome. Egypt J Intern Med.

